# The oligodendroglia cytoskeleton in health and disease

**DOI:** 10.1186/2193-1801-4-S1-L15

**Published:** 2015-06-12

**Authors:** Christiane Richter-Landsberg

**Affiliations:** University of Oldenburg, Molecular Neurobiology, Germany

**Keywords:** Oligodendrocytes, microtubules, neurodegenerative diseases

Oligodendrocytes, the myelin forming cells of the CNS, enwrap neuronal axons and form multilamellar myelin sheets. They are derived from oligodendrocyte precursor cells which migrate from the subventricular zone into the different regions of the brain. Differentiation from the early progenitor to the mature multiprocessed oligodendrocyte is characterized by different morphological stages. To support cell morphology and establish and maintain the myelin membrane, an intact, spatially organized cytoskeleton with dynamic properties is essential. In particular microtubules and their associated proteins play an important role. A variety of microtubule binding proteins, including tau, are present in oligodendrocytes. Oligodendrocytes in culture express all six isoforms of tau which are developmentally regulated. Tau proteins are present in immature and mature oligodendrocytes and specifically prominent in the branching points of the cellular processes. Downregulation of tau impairs cell differentiation and the process of early myelination. In neurodegenerative diseases collectively termed tauopathies, fibrillary tau accumulations occur not only in neurons but also in glia. Tau positive coiled bodies originating in oligodendrocytes are characteristic for the brains of patients with frontotemporal dementias, such as corticobasal degeneration and progressive supranuclear palsy. These aggregates are further characterized by the presence of heat shock proteins and ubiquitin, indicating that stress situations are causally related to the pathogenesis. In this respect, proteasomal dysfunctions have been discussed to be involved in neurodegenerative disorders and the aging process. Data on the consequences of proteolytic stress in oligodendrocytes and the protein aggregation process will be presented. Our data demonstrate that an intact cytoskeleton is essential for cellular defense mechanisms.

